# Small fiber involvement is independent from clinical pain in late-onset Pompe disease

**DOI:** 10.1186/s13023-022-02327-4

**Published:** 2022-04-27

**Authors:** Elena K. Enax-Krumova, Iris Dahlhaus, Jonas Görlach, Kristl G. Claeys, Federica Montagnese, llka Schneider, Dietrich Sturm, Tanja Fangerau, Hannah Schlierbach, Angela Roth, Julia V. Wanschitz, Wolfgang N. Löscher, Anne-Katrin Güttsches, Stefan Vielhaber, Rebecca Hasseli, Lea Zunk, Heidrun H. Krämer, Andreas Hahn, Benedikt Schoser, Angela Rosenbohm, Anne Schänzer

**Affiliations:** 1 Department of Neurology, BG University Hospital Bergmannsheil, Ruhr-University, Bochum, Germany; 2 Heimer-Institute for Muscle Research, BG University Hospital Bergmannsheil, Ruhr-University, Bochum, Germany; 3 Institute of Medical Informatics, Charité – Universitätsmedizin Berlin, Berlin, Germany; 4 Institute of Neuropathology, Justus Liebig University Giessen, Arndstr.16, 35392 Giessen, Germany; 5 Department of Neurology, University Hospitals Leuven, Leuven, Belgium; 6 Laboratory for Muscle Diseases and Neuropathies, Department of Neurosciences, KU Leuven, Leuven, Belgium; 7 Friedrich-Baur-Institute, Department of Neurology, LMU University Munich, Munich, Germany; 8 Department of Neurology, Martin Luther University Halle-Wittenberg, Halle, Germany; 9 Department of Neurology, University of Ulm, Ulm, Germany; 10 Department of Neurology, Medical University Innsbruck, Innsbruck, Austria; 11 Department of Neurology, Otto-Von-Guericke University, Magdeburg, Germany; 12 Department of Rheumtaology and Clinical Immunology, Campus Kerkhoff, Justus-Liebig University, Giessen, Germany; 13 Department of Neurology, Justus Liebig University, Giessen, Germany; 14 Department of Child Neurology, Justus Liebig University, Giessen, Germany; 15 Department of Neurology, St Georg Hospital, Leipzig, Germany

**Keywords:** Late onset Pompe disease, Small nerve fiber, Pain, Skin biopsy, Intraepidermal nerve fiber density

## Abstract

**Background:**

Pain occurs in the majority of patients with late onset Pompe disease (LOPD) and is associated with a reduced quality of life. The aim of this study was to analyse the pain characteristics and its relation to a small nerve fiber involvement in LOPD patients.

**Methods:**

In 35 patients with LOPD under enzyme replacement therapy without clinical signs of polyneuropathy (19 females; 51 ± 15 years), pain characteristics as well as depressive and anxiety symptoms were assessed using the PainDetect questionnaire (PDQ) and the hospital anxiety and depression scale (HADS), respectively. Distal skin biopsies were analysed for intraepidermal nerve fiber density (IENFD) and compared to age- and gender-matched reference data. Skin biopsies from 20 healthy subjects served as controls to assure validity of the morphometric analysis.

**Results:**

Pain was reported in 69% of the patients with an average intensity of 4.1 ± 1.1 on the numeric rating scale (NRS; anchors: 0–10). According to PDQ, neuropathic pain was likely in one patient, possible in 29%, and unlikely in 67%. Relevant depression and anxiety symptoms occurred in 31% and 23%, respectively, and correlated with pain intensity. Distal IENFD (3.98 ± 1.95 fibers/mm) was reduced in 57% of the patients. The degree of IENFD reduction did not correlate with the durations of symptoms to ERT or duration of ERT to biopsy.

**Conclusions:**

Pain is a frequent symptom in treated LOPD on ERT, though a screening questionnaire seldom indicated neuropathic pain. The high frequency of small nerve fiber pathology in a treated LOPD cohort was found regardless of the presence of pain or comorbid risk factors for SFN and needs further exploration in terms of clinical context, exact mechanisms and when developing novel therapeutic options for LOPD.

**Supplementary Information:**

The online version contains supplementary material available at 10.1186/s13023-022-02327-4.

## Introduction

Pompe disease (OMIM 232300) is an autosomal recessive disease due to mutations in the α-1,4-glucosidase gene (*GAA*) and consequent lysosomal α-glucosidase deficiency [1]. More than 500 variants of *GAA* genetic mutations have been identified and the clinical phenotypes show a high variability without clear relation to a certain genotype [2–8]. In patients with infantile onset Pompe disease (IOPD) and late onset Pompe disease (LOPD), enzyme replacement treatment (ERT) with recombinant GAA reduces muscle weakness, respiratory insufficiency, and increases life expectancy [9–12]. However, despite ongoing ERT patients can develop new phenotypes involving the central and peripheral nervous system [13–17].

Pain occurs in 50–80% of patients with LOPD, especially in ERT-naïve patients, and is associated with a reduced quality of life, and higher levels of anxiety and depression [18–23]. Patients mostly report exhausting pain in the limb girdle muscles and lower back, but also burning pain in the legs. Pain medication often does not lead to sufficient pain relief [18, 23]. Both nociceptive and neuropathic pain components might be relevant for pain symptoms in Pompe disease, requiring different treatment regimes. Small nerve fiber damage is associated with chronic pain and small nerve fiber involvement has also been suggested in patients with IOPD and LOPD [15, 24–27].

The aim of this study was to assess the pain prevalence and characteristics in a cohort of patients with LOPD, and to analyse its association to small nerve fiber density in skin biopsies as well as to clinical parameters, including anxiety and depressive symptoms.

## Methods

### Subject and samples

From 2017 to 2020, patients with LOPD receiving ERT were recruited in six German, one Belgian, and one Austrian neuromuscular centres and were included in the study if a Pompe disease was confirmed by reduced GAA enzyme activity and genetic testing. Patients were excluded if they had clinical signs of large fiber involvement detected by nerve conduction studies. Healthy subject without any signs for neuropathic pain or medical history of risk factors for SFN served as controls.

### PainDETECT questionnaire (PDQ)

The painDETECT Questionnaire (PDQ) is a validated screening tool that discriminates between neuropathic and nociceptive pain [28]. At the time of skin biopsy, patients rated their current, worse, and average pain intensity over the last 4 weeks on an 11-point numeric rating scale (NRS; 0 = “no pain” to 10 = “worst imaginable pain”). PDQ-scores ≥ 19 indicated a neuropathic pain (NP) component, for scores ≤ 12 NP was considered unlikely, and in the case of scores between 13 and 18, NP was considered uncertain [28].

### Hospital anxiety and depression scale (HADS)

On the same day as the skin biopsy, the Hospital Anxiety and Depression Scale (HADS) was assessed in the validated German version, including two scores for the degree of depression (HADS-D) and anxiety (HADS-A), respectively (abnormal: score > 7 for each) [29, 30].

### Skin punch biopsy

To determine the intraepidermal nerve fiber density (IENFD) 3 mm skin punch biopsies were taken 10 cm above the lateral malleolus (distal) in all included patients. Additionally, from twenty healthy controls skin biopsies were obtained from the same location assure validity of the morphometric analysis. Skin samples were fixed with Zamboni, washed in PBS, transferred to 10% sucrose, and stored at − 80 °C in a freezer until further use. From each biopsy, 50 µm-thick frozen sections were stained using a free-floating protocol with primary antibody anti-protein gene product (PGP 9.5, 1:1000, Zytomed), and secondary antibody goat anti-rabbit Alexa Fluor 488 (1:1000, Thermo Fisher Scientific) [24, 31]. Sections were mounted with DAPI Fluorshield (Abcam) and examined with a Leica DM 2000 fluorescence microscope (Leica Microsystems, Wetzlar, Germany) at magnification × 40 with a Leica DFC450C camera, Leica Application-Suite Version 4.7.1,). The investigators (JG and AS) were blinded to the clinical data during the morphologic analysis.

### Quantifying intraepidermal nerve density (IENFD)

According to published counting recommendations, IENFD was determined in at least six sections from each biopsy and was compared to published age and gender-related reference values [32]. In addition, IENFD were z-transformed as ${z}_{individual}=\frac{{IENFD}_{individual}-{IENFD}_{reference}}{{SD}_{reference}}$ using the reference values specific for age and gender. IENFD was either considered “reduced” if below the 5th percentile of the reference data.

### Assessment of the corneal innervation

Corneal confocal microscopy (CCM) was available in only one of the study centers and as a proof of concept four patients underwent additionally a detailed assessment of the corneal innervation by CCM. Four CCM images from the left eye of each patient were manually analyzed using an established software (CCMetrics, Version 2.0, M.A. Dabbah, University of Manchester) to visualize the corneal subbasal nerve plexus. The average corneal nerve fiber length (CNFL, mm/mm^2^), density (CNFD, nerves/mm^2^), and branch density (CNBD, branch points) were analyzed and compared to normative data [33]. Image quality was assessed and confirmed as previously described [34].

### Statistical analysis

The correlation between pain scores and HADS subscores, z-scores and further clinical data were analysed by calculating the Pearson’s correlation coefficient to assess possible linear associations between the variables. Differences between patients with and without pain regarding the HADS subscores and differences between patients and controls regarding IENFD were analysed using Mann–Whitney U test. The distribution of nominal variables was tested using the chi-squared test. To assesses whether pain scores, duration from symptoms onset to biopsy (symptoms to biopsy), duration from symptoms onset to onset ERT therapy (symptoms to ERT) and duration from onset ERT therapy and biopsy (ERT to biopsy) differ between patients with normal or reduced IENFD were submitted to analysis of multivariate variance (multivariate ANOVA) using the standard ‘manova’ function of the R software package. Statistical analysis was performed using IBM SPSS Statistics, version 24 and R software package (version 3.6.3; http://CRAN.R-project.org/ (R Development Core Team, 2008)). P-values < 0.05 were regarded as significant.

## Results

### Demographic and clinical data

Thirty-five patients with LOPD on regular ERT with recombinant alpha glucosidase (Myozyme® 20 mg/kg of body weight administered every 2 weeks) were included in the study. All patients were compound heterozygote for GAA, with the most prevalent mutation being c-32-13 T > G in 88%. In one patient, the exact genetic results were missing (Table 1). Eight patients had a comorbidity, which was regarded as a low risk factor for SFN (see Table 1). Detailed demographic and clinical data are listed in Additional file 1: Table S1.

**Table 1:** Summary of data in patients with LOPD (n = 35) included in the study

**Gender**	
Female [number (%)]	19 (54%)
Male [number (%)]	16 (46%)
**Age at** [years, mean (range)]	
Skin biopsy	50.3 (18–74)
Symptoms	34.9 (3–72)
Diagnosis	42.3 (4–74)
Start ERT	43.8 (4–74)
Duration onset symptoms-time of biopsy	15.3 (1–44)
Duration onset ERT—time of biopsy	6.5 (0–14)
**Genetic mutation** (34/35)	
c.-32-13T > G [n (%)]	30 (88%)
c.45T > G [n (%)]	3 (9%)
Others	1 (3%)
**GAA residual level**—reduction in % (23/35) [average (range)]	89% (73–99)
**Risk for PNP** [number (%)]	8 (23%)
Diabetes mellitus type 2	3
Frequent alcohol intake	2
Cobalamin or/and ferritin deficiency	3
**IENFD** (35/35)	
Reduced [number (%)]	20 (57%)
**Pain within the last 4 weeks** (35/35)	24 (69%)
Current pain intensity (NRS 0–10) [mean ± SD (range)] Average pain intensity (NRS 0–10) [mean ± SD (range)] Maximal pain intensity (NRS 0–10) [mean ± SD (range)]	2.8 ± 2.6 (0–9) 4.1 ± 1.1 (1–7) 6.2 ± 2.2 (2–10)
Pain attacks without pain between them [number (%)] Persistent pain with slight fluctuations [number (%)] Persistent pain with pain attacks [number (%)] Pain attacks with pain between them [number (%)]	13 (54%) 8 (33%) 2 (8%) 1 (4%)
Neuropathic pain likely [number (%)] Neuropathic pain component unclear [number (%)] Neuropathic pain unlikely [number (%)]	1 (3%) 7 (20%) 16 (46%)
**HADS score** (35/35)	
Relevant anxiety and/or depressive symptoms [number (%)]	12 (34%)
Relevant depression symptoms [number (%)]	8 (23%)
Relevant anxiety symptoms [number (%)]	11 (31%)

ERT, enzyme replacement therapy; GAA, acid alpha glucosidase; PNP, polyneuropathy; IENFD, intraepidermal nerve fiber density; NRS, numeric rating scale; HADS, hospitality anxiety and depression scale

### Pain characteristics

**Fig. 1:**
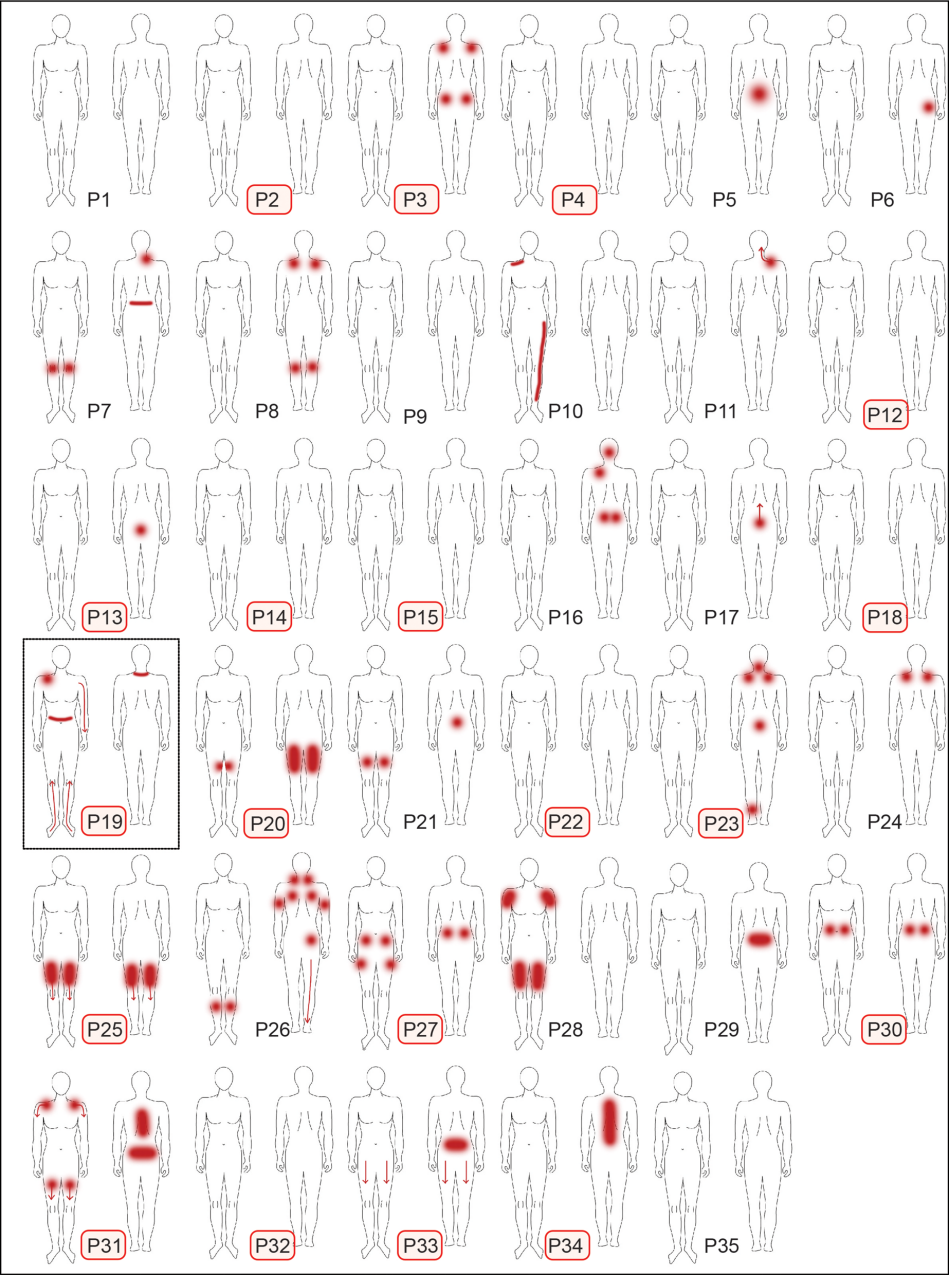
Pain distribution in patients with LOPD: twenty-four (69%) of the patients reported pain including axial and joint pain in most of the cases. Only on patient (P19) reported neuropathic pain in the distal legs (large black rectangular frame). Reduction of IENFD was detected in 57% of the patients (small red rectangular frames)

Twenty-four patients (69%) reported pain within the last 4 weeks with an average intensity of 4.1 ± 1.1 on the NRS with the anchors 0–10 (0: no pain; 10: worst pain imaginable) including axial and joint pain in most cases, and the distal leg only in one case (Fig. 1, Table 1). Pain attacks with or without continuous pain in between was the leading symptom in 58% of the patients, while persistent pain with slight or stronger fluctuations was reported in 42% of the patients (Table 1). The PDQ score suspected an NP component in only one patient (P19) (Fig. 1). Six of the eight patients with a risk factor for developing a polyneuropathy reported pain, though the PDQ-score did not suspect a likely NP in any of them (Table 1, Additional file 2: Table S2). The PDQ score significantly correlated with the current (r = 0.47, p < 0.05), average (r = 0.71, p < 0.001) and maximal pain intensity (r = 0.59, p < 0.01) with higher PDQ scores in cases of higher pain intensity.

### Anxiety and depression symptoms

**Fig. 2:**
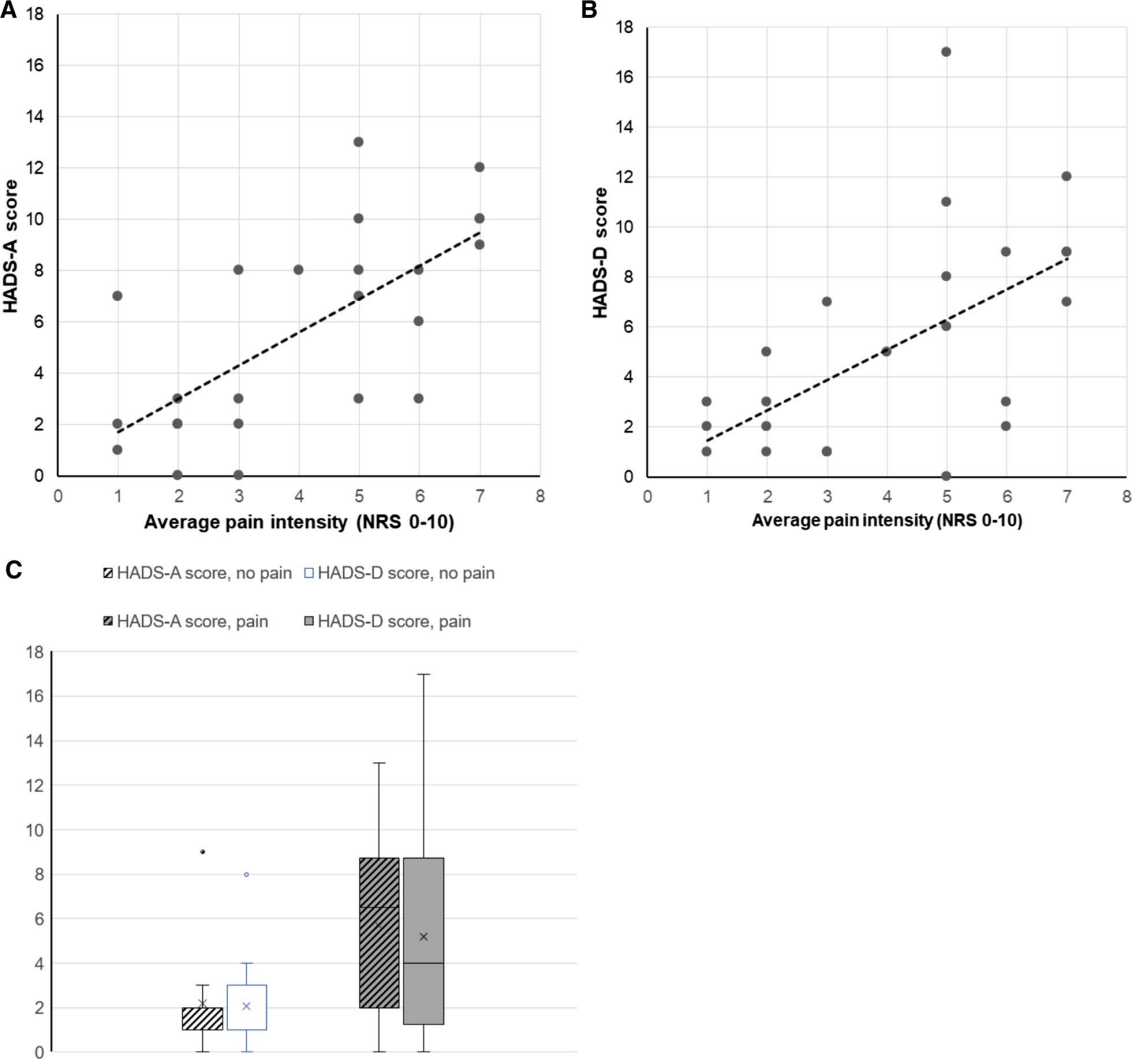
Correlation between the pain intensity and the **A** degree of anxiety symptoms (HADS-A score) and the **B** degree of depressive symptoms (HADS-D score). **C** Distribution of the HADS-A and HADS-D scores in patients with vs. without pain (x = mean)

Twelve patients (34%) reported relevant anxiety and/or depression symptoms according to HADS (Table 1). Only one of the elven patients with abnormal HADS-A scores and one of the eight patients with abnormal HADS-D scores were pain free. The HADS-A and the HADS-D scores showed a highly significant correlation with each other (r = 0.84, p < 0.001), and both correlated significantly (p < 0.001) with the current (r = 0.56 and r = 0.57, respectively), average (r = 0.59 and r = 0.52, respectively), and maximal pain intensity (r = 0.70 and r = 0.61, respectively) (Fig. 2A, B). The HADS-A and the HADS-D scores were higher in the subgroup of patients with pain versus patients without pain, though the difference was not significant (Fig. 2C). Detailed data can be found in the Additional file 2: Table S2.

### Quantifying intraepidermal nerve density (IENFD)

**Fig. 3:**
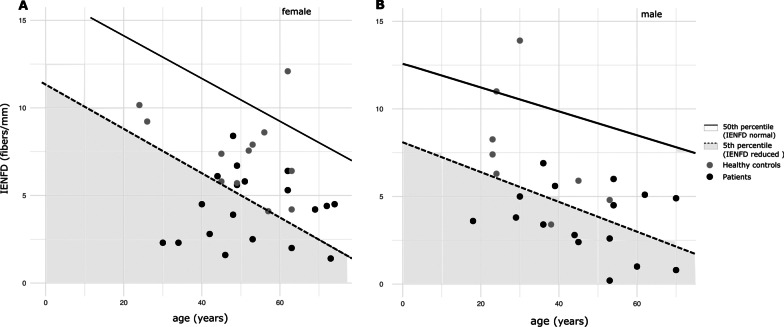
Scatter plot showing intraepidermal nerve fiber density (IENFD) values in **A** female patients (black dots) and healthy controls (grey dots) and **B** male patients (black dots) and healthy controls (grey dots). Solid line depict 50th and dotted line 5th percentiles of normative values from Lauria et al. Values below the 5th percentile (grey area) are considered as reduced

The average IENFD of patients was 3.98 ± 1.95 fibers/mm (zscore: − 2.12 ± 0.825), whereas the average IENFD of healthy subjects was 7.50 ± 1.95 fibers/mm (zscore: − 1.09 ± 1.02), the latter being in the range of published normative data. Patients differed significantly (p < 0.05) from controls regarding their IENFD confirming the overall loss of fibres in LOPD patients. Compared to an established age- and gender-matched reference data IENFD was considered reduced (< 5th percentile) in 57% of the patients (Fig. 3 A and B, Additional file 3: Table S3). The only one patient with increased PDQ score suggestive of neuropathic pain had also reduced IENFD at the lower leg. Five of the eight patients with a risk factor for developing a polyneuropathy presented with a reduced IENFD at the lower leg.

**Fig. 4:**
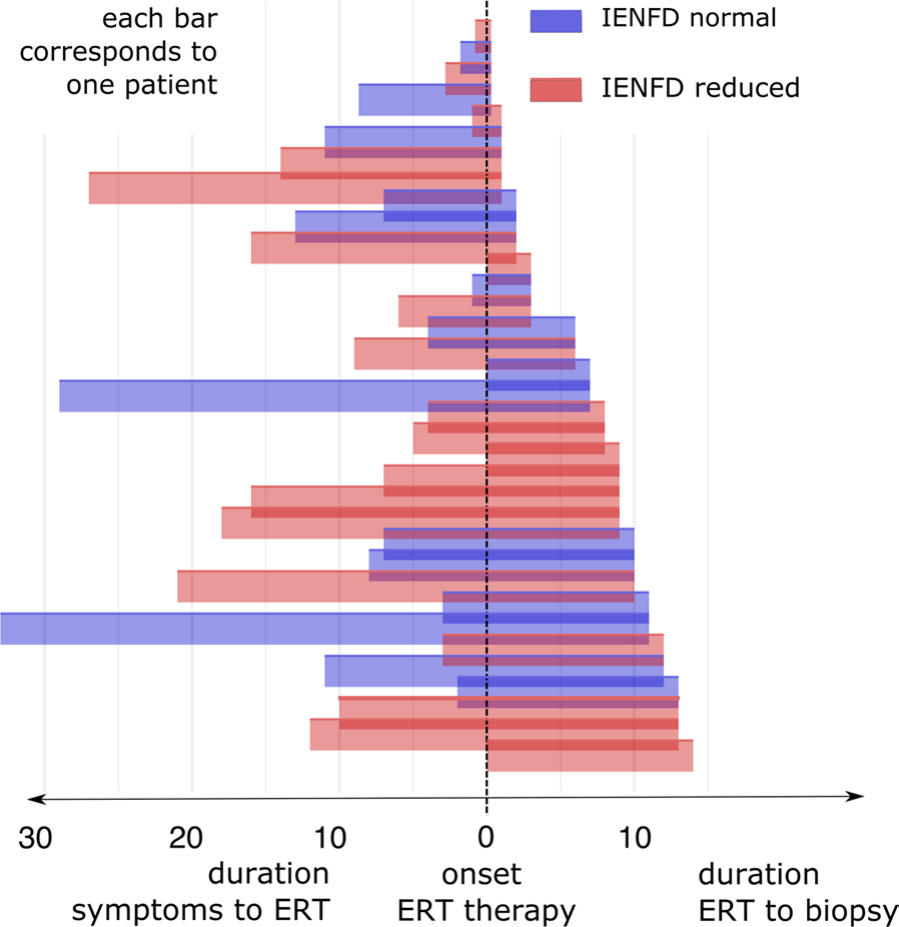
Bar chart showing time periods (duration of symptoms to onset of ERT therapy, onset of ERT therapy to biopsy) and patients with reduced (red) and normal (blue) intraepidermal nerve fiber density (IENFD) at time of biopsy (right end of bar). Each patient is represented by a bar, with the sum of the left and right values representing the total time from symptom onset to the time of biopsy. The distribution of patients with reduced fiber density is evenly distributed across the cohort

Patients displaying reduced IENFD at biopsy were on average 8.6 ± 6.55 years after symptom onset without ERT therapy and 7.93 ± 4.77 years on ERT therapy. For patients with normal IENFD mean duration from the onset of symptoms to ERT therapy was 9.33 ± 6.33 years and duration from the onset of ERT therapy to biopsy was 8.6 ± 6.55 years. Regardless of the different times before and after the onset of ERT, reduced IENFD occurred across all time periods and no visible pattern was detected (Fig. 4).

**Fig. 5:**
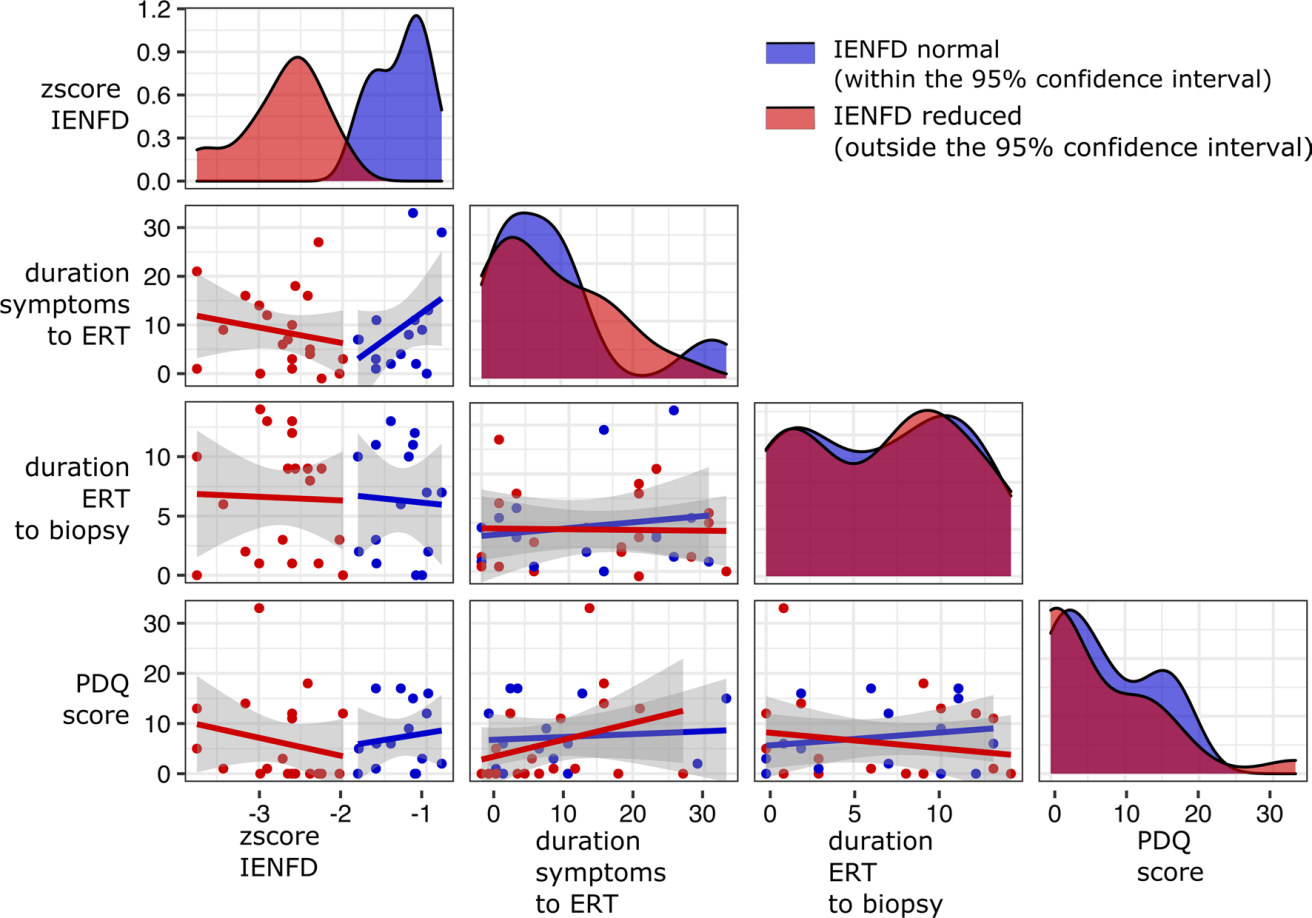
Correlation scatter plot matrix of fiber densities (z-score IENFD) and clinical data. i.e. time between symptoms onset and start of ERT (duration symptoms to ERT), start of ERT therapy to time of biopsy (duration ERT to biopsy) and PDQ-score in patients with reduced (red) and normal (blue) fiber densities. The lines indicate a linear fit (and confidential intervals) for visual guidance of possible correlations. The diagonal shows the data distribution as probability density function

Although the degree of fiber reduction did not correlate with the durations and the main effects of MANOVA did not reach significance, patients in the group of reduced IENFD showed a trend towards higher pain scores associated with a higher nerve fiber loss (Fig. 5).

### Corneal confocal microscopy

Analyzing CCM, 3 out of 4 examined patients showed an abnormal reduction of at least one of the corneal nerve parameters, most often of the CNFL, corresponding to a reduced IENFD at the lower leg in two of them; one patients with normal CCM findings had a reduced IENFD at the lower leg (Table 2, Additional file 5: Figure S1).

**Table 2:** Average scores of corneal nerve parameters

P. Nr	CNFD	CNBD	CNFL	CCM Appraisal	Distal IENFD
4	15,62	**12,50**	**10,85**	Abnormal	Reduced
3	32,81	21,87	16,72	Normal	Reduced
5	14,06	20,31	**9,22**	Abnormal	Normal
18	17,19	26,56	**12,76**	Abnormal	Reduced

Pathological results illustrated in bold.

CNFD, corneal nerve fiber density; CNBD, corneal nerve branch density; CNFL, corneal nerve fiber length; CCM, corneal confocal microscopy; IENFD, intraepidermal nerve fiber density

## Discussion

The present multicentre study evaluated the correlation between peripheral skin innervation and clinical parameters in 35 patients with LOPD during ongoing ERT. The multimodal phenotyping included morphometric skin biopsy analysis and self-reported assessment of pain, depression and anxiety symptoms based on validated questionnaires. Pain was a relevant symptom in over two-thirds of our patients. Furthermore, up to one-third of the patients reported relevant anxiety or depression symptoms, correlating with their pain intensity. A manifest reduction of the skin innervation in the distal lower limb was found in 57% of the patients based on published normative dataset, regardless of the presence of pain or comorbid risk factors for SFN [32].

### Pain characteristics

Pain can be a common and debilitating symptom in Pompe disease [18–23]. With a prevalence of 69% for any pain in the last 4 weeks in our cohort, our results are well within the range of previous reports, which varied between 45% within the last 24 h and 88% within the last 7 days [18, 23]. In contrast to the reported higher pain intensity in ERT naïve patients [20], the pain intensity in our LOPD cohort under ERT was only mild to moderate, in line with previous studies [18]. Pain intensity correlated with the degree of anxiety and depression symptoms, which have been also previously reported to be increased in patients with LOPD [18]. These findings indicate a plausible explanation for the previously reported reduced quality of life of LOPD patients can be eplained [20].

Most patients in our study described pain attacks and persistent pain in the axial and limb girdle area, similar to previous reports [18, 20]. This pain distribution is unlike the classical clinical presentation of length-dependent small fiber neuropathy [35, 36]. Though, non-length dependent small fiber neuropathy can present with various phenotypes, which has to be considered in the diagnostic workout and also for future studies [37]. Notably in our study, the self-reported pain characteristics indicated that the pain was unlikely to be neuropathic, and thus we can assume that other pain mechanisms are also relevant in LOPD. This is in line with the results of a 12-week training program aiming at increasing aerobic fitness and muscle strength, which was able to reduce the pain prevalence from 56 to 21% in a cohort of LOPD [22]. Therefore, musculoskeletal nociceptive pain might be more relevant in LOPD patients.

### Morphometric findings

In our study, despite ongoing ERT, the IENFD was reduced in a large number of patients compared to age- and gender-matched reference data [32]. The IENFD reduction did not correlate with most clinical data, e.g. duration of symptoms to ERT or duration from ERT start to biopsy. However, the magnitude of IENFD reduction in the group of patients with reduced IENFD was associated with higher pain ratings, though the correlation was not significant in our cohort.

Until now, small fiber involvement in IOPD and LOPD was evaluated based on self-assessment using the small fiber neuropathy screening list [27]. This screening tool was validated only for sarcoidosis [38]. Its specificity for detecting small fiber involvement in Pompe disease might be hampered due to inclusion of items that might be positive also due to the affection of skeletal and smooth muscles in Pompe disease, such as cramps, oro-gastrointestinal, and urinary tract alterations [39]. In contrast to self-reported questionnaire, analyzing nerve fibers in distal skin biopsies is a validated approach fore direct assessment of the damage at the distal part of the nerve axons.

Four patients underwent additionally assessment of corneal innervation using CCM and three of these patients presented additionally with at least one abnormal CCM parameter. A reduced corneal innervation has been reported in patients with peripheral neuropathy of different origin, such as metabolic, toxic, lysosomal, or infectious diseases [40–45]. Although CCM was performed only in a small subgroup, its results support the skin biopsy findings of a reduced peripheral innervation in LOPD under ERT.

### Pathophysiological consideration

In Pompe disease glycogen storage can occur in all kinds of different cell types including cells of the peripheral and nervous system [39, 46]. The reduction of small dermal nerve fibers as shown in our study, might result from pathological glycogen storage and cell dysfunction in the peripheral nervous system e.g. Schwann cells or dorsal root ganglion cells. Glycogen deposits were also found in smooth muscle vascular cells and might be associated with cutaneous microcirculation dysfunction [15, 47, 48]. However, glycogen deposits in skin biopsies have described in the errector pili muscle in Pompe patients, but other dermal structures, e.g. sweat glands and vasculature, have not been analyzed in detail [49]. In animal model of GAA deficient mice both a reduction of sensory dorsal root ganglion cells with axonal damage of sensory neurons as well as glycogen deposits in Schwann cells with signs of nerve demyelination have been reported [50, 51]. Further studies are needed to differentiate whether sensory and/or autonomic small fibers alone or peripheral nerves in general are affected in LOPD.

### Strengths and limitations

Strengths of our study are the large size of the patient cohort despite the rare disease, as well as the assessment of both self-reported validated questionnaires and morphometric data. Further, patients with polyneuropathy and manifest involvement of the large fibers have been excluded.

One limitation of our study is that assessment of the small fiber function (detailed clinical bedside sensory testing, quantitative thermal testing, sudomotor tests, autonomic tests) has not been performed. This prevents final conclusions about the presence of small fiber neuropathy or neuropathic pain per definition [36, 52, 53]. Further, we did not include ERT-naïve patients, though can be expected to report stronger pain [22]. Another challenge is that CCM assessment was available only from 4 patients; thus, final appraisal on the involvement of corneal nerve fibers in LOPD and its correlation with distal skin innervation is not possible. Future longitudinal studies should evaluate any abnormalities of small fiber innervation both in the skin and in the corneal subbasal plexus, which would help to assess treatment effects during ongoing ERT also in a non-invasive manner.

## Conclusion

To summarize, pain is a common symptom in LOPD that interferes with psychological aspects and the patients’ quality of life. We found reduced small nerve fiber density in a large number of LOPD patients under ERT. Thus, our results indicate that the peripheral nervous system may represent another system affected in Pompe disease, which is in line with few previous reports on neuropathy in Pompe disease [15, 16]. However, the pain characteristics and distribution did not indicate of neuropathic pain or SFN in our cohort of patients with LOPD. Future studies including standardized longitudinal assessments of LOPD regarding small nerve fiber pathology and function, as recently presented for SFN [54] may give further information both on the underlying mechanisms of small nerve fiber degeneration and pain generation and on the disease’s progress, thus potentially contributing to personalized treatment.

## Supplementary Information

**Additional file 1:** Table S1: Clinical and demographic findings in 35 patients with LOPD

**Additional file 2:** Table S2: Self-reported data on pain, anxiety and depression symptoms in 35 patients with LOPD

**Additional file 3:** Table S3: Morphometric analysis of skin biopsies from 35 patients with LOPD

**Additional file 4:** Table S4: Morphometric analysis of skin biopsies from twenty healthy controls

**Additional file 5:** Fig. S1: Four patients with LOPD underwent detailed assessment of the corneal innervation by corneal confocal microscopy (CCM): While P3 showed a reduction of small nerve fibers only in the distal skin biopsy, P5 presented with abnormalities only in the corneal innervation.

## Data Availability

Data are available in the supplementary tables.

## Abbreviations

CK: Creatine kinase
CCM: Corneal confocal microscopy
DBT: Dried blood test
ERT: Enzyme replacement therapy
GAA: Glucosidase alpha acid
HADS: Hospitality anxiety depression scale
PDQ: Pain detection questionnaire
NRS: Numeric rating scale
IENFD: Intraepidermal nerve fiber density
IOPD: Infantile onset Pompe disease
y: Years
QST: Quantitative sensory testing
LOPD: Late onset Pompe disease
N.S.: Not significant
NP: Neuropathic pain
SFN: Small fiber neuropathy

## References

[1] Hoefsloot LH, Hoogeveen-Westerveld M, Reuser AJ, Oostra BA (1990). Characterization of the human lysosomal alpha-glucosidase gene. Biochem J.

[2] De Filippi P, Saeidi K, Ravaglia S, Dardis A, Angelini C, Mongini T, Morandi L, Moggio M, Di Muzio A, Filosto M (2014). Genotype-phenotype correlation in Pompe disease, a step forward. Orphanet J Rare Dis.

[3] Montagnese F, Barca E, Musumeci O, Mondello S, Migliorato A, Ciranni A, Rodolico C, De Filippi P, Danesino C, Toscano A (2015). Clinical and molecular aspects of 30 patients with late-onset Pompe disease (LOPD): unusual features and response to treatment. J Neurol.

[4] Kroos MA, Pomponio RJ, Hagemans ML, Keulemans JL, Phipps M, DeRiso M, Palmer RE, Ausems MG, Van Beek NA, Van Diggelen OP (2007). Broad spectrum of Pompe disease in patients with the same c.-32–13T>G haplotype. Neurology.

[5] Kroos M, Hoogeveen-Westerveld M, van der Ploeg A, Reuser AJ (2012). The genotype-phenotype correlation in Pompe disease. Am J Med Genet C Semin Med Genet.

[6] Kroos M, Hoogeveen-Westerveld M, Michelakakis H, Pomponio R, Van der Ploeg A, Halley D, Reuser A (2012). Update of the pompe disease mutation database with 60 novel GAA sequence variants and additional studies on the functional effect of 34 previously reported variants. Hum Mutat.

[7] Sampaolo S, Esposito T, Farina O, Formicola D, Diodato D, Gianfrancesco F, Cipullo F, Cremone G, Cirillo M, Del Viscovo L (2013). Distinct disease phenotypes linked to different combinations of GAA mutations in a large late-onset GSDII sibship. Orphanet J Rare Dis.

[8] Kulessa M, Weyer-Menkhoff I, Viergutz L, Kornblum C, Claeys KG, Schneider I, Plöckinger U, Young P, Boentert M, Vielhaber S (2020). An integrative correlation of myopathology, phenotype, and genotype in late onset Pompe disease. Neuropathol Appl Neurobiol.

[9] Hahn A, Schänzer A. Long-term outcome and unmet needs in infantile-onset Pompe disease. Ann Transl Med*.* 2019;283. 10.21037/atm.2019.04.70 PMC6642934 31392195

[10] van der Ploeg A, Carlier PG, Carlier RY, Kissel JT, Schoser B, Wenninger S, Pestronk A, Barohn RJ, Dimachkie MM, Goker-Alpan O (2016). Prospective exploratory muscle biopsy, imaging, and functional assessment in patients with late-onset Pompe disease treated with alglucosidase alfa: the EMBASSY Study. Mol Genet Metab.

[11] Van den Hout JM, Kamphoven JH, Winkel LP, Arts WF, De Klerk JB, Loonen MC, Vulto AG, Cromme-Dijkhuis A, Weisglas-Kuperus N, Hop W (2004). Long-term intravenous treatment of Pompe disease with recombinant human alpha-glucosidase from milk. Pediatrics.

[12] Schoser B, Stewart A, Kanters S, Hamed A, Jansen J, Chan K, Karamouzian M, Toscano A (2017). Survival and long-term outcomes in late-onset Pompe disease following alglucosidase alfa treatment: a systematic review and meta-analysis. J Neurol.

[13] McIntosh PT, Hobson-Webb LD, Kazi ZB, Prater SN, Banugaria SG, Austin S, Wang R, Enterline DS, Frush DP, Kishnani PS (2018). Neuroimaging findings in infantile Pompe patients treated with enzyme replacement therapy. Mol Genet Metab.

[14] Ebbink BJ, Poelman E, Aarsen FK, Plug I, Regal L, Muentjes C, van der Beek N, Lequin MH, van der Ploeg AT, van den Hout JMP (2018). Classic infantile Pompe patients approaching adulthood: a cohort study on consequences for the brain. Dev Med Child Neurol.

[15] Schänzer A, Görlach J, Claudi K, Hahn A. Severe distal muscle involvement and mild sensory neuropathy in a boy with infantile onset Pompe disease treated with enzyme replacement therapy for 6 years. Neuromuscul Disord*.* 2019;477–482. 10.1016/j.nmd.2019.03.004 31101460

[16] Lamartine SMM, Remiche G (2019). Late-onset Pompe disease associated with polyneuropathy. Neuromuscul Disord.

[17] Toscano A, Rodolico C, Musumeci O (2019). Multisystem late onset Pompe disease (LOPD): an update on clinical aspects. Ann Transl Med.

[18] Güngör D, Schober AK, Kruijshaar ME, Plug I, Karabul N, Deschauer M, van Doorn PA, van der Ploeg AT, Schoser B, Hanisch F (2013). Pain in adult patients with Pompe disease: a cross-sectional survey. Mol Genet Metab.

[19] Hamed A, Curran C, Gwaltney C, DasMahapatra P (2019). Mobility assessment using wearable technology in patients with late-onset Pompe disease. NPJ Digit Med.

[20] Schoser B, Bilder DA, Dimmock D, Gupta D, James ES, Prasad S. The humanistic burden of Pompe disease: are there still unmet needs? A systematic review. BMC Neurol. 17 10.1186/s12883-017-0983-2 PMC5700516 29166883

[21] Gesquiere-Dando A, Attarian S, Maues De Paula A, Pouget J, Salort-Campana E (2015). Fibromyalgia-like symptoms associated with irritable bowel syndrome: a challenging diagnosis of late-onset Pompe disease. Muscle Nerve.

[22] Favejee MM, van den Berg LE, Kruijshaar ME, Wens SC, Praet SF, Pim Pijnappel WW, van Doorn PA, Bussmann JB, van der Ploeg AT (2015). Exercise training in adults with Pompe disease: the effects on pain, fatigue, and functioning. Arch Physl Med Rehabil.

[23] Karabul N, Kruijshaar ME, Schober A, Güngör D, Hanisch F (2014). Pain in adult patients with Pompe disease. Mol Genet Metab Rep.

[24] Görlach J, Amsel D, Kölbel H, Grzybowsky M, Rutsch F, Schlierbach H, Vanlander A, Pogatzki-Zahn E, Habig K, Garkisch S (2020). Diagnostic utility of small fiber analysis in skin biopsies from children with chronic pain. Muscle Nerve.

[25] Sopacua M, Hoeijmakers JGJ, Merkies ISJ, Lauria G, Waxman SG, Faber CG (2019). Small fibre neuropathy: expanding the clinical pain universe. J Peripher Nerv Syst.

[26] Üçeyler N (2016). Small fiber pathology–a culprit for many painful disorders?. Pain.

[27] Hobson-Webb LD, Austin SL, Jain S, Case LE, Greene K, Kishnani PS (2015). Small-fiber neuropathy in pompe disease: first reported cases and prospective screening of a clinic cohort. Am J Case Rep.

[28] Freynhagen R, Baron R, Gockel U, Tolle TR (2006). painDETECT: a new screening questionnaire to identify neuropathic components in patients with back pain. Curr Med Res Opin.

[29] Hinz A, Schwarz R (2001). Anxiety and depression in the general population: normal values in the Hospital Anxiety and Depression Scale. Psychother Psychosom Med Psychol.

[30] Zigmond AS, Snaith RP (1983). The hospital anxiety and depression scale. Acta Psychiatr Scand.

[31] Kennedy WR, Wendelschaefer-Crabb G, Polydefkis M, McArthur JC, Dyck PJ, Thomas PK (2005). Pathology and quantitation of cutaneous innervation. Peripheral neuropathy.

[32] Lauria G, Bakkers M, Schmitz C, Lombardi R, Penza P, Devigili G, Smith AG, Hsieh ST, Mellgren SI, Umapathi T (2010). Intraepidermal nerve fiber density at the distal leg: a worldwide normative reference study. J Peripher Nerv Syst.

[33] Tavakoli M, Ferdousi M, Petropoulos IN, Morris J, Pritchard N, Zhivov A, Ziegler D, Pacaud D, Romanchuk K, Perkins BA (2015). Normative values for corneal nerve morphology assessed using corneal confocal microscopy: a multinational normative data set. Diabetes Care.

[34] Sturm D, Vollert J, Greiner T, Rice ASC, Kemp H, Treede RD, Schuh-Hofer S, Nielsen SE, Eitner L, Tegenthoff M (2019). Implementation of a quality index for improvement of quantification of corneal nerves in corneal confocal microcopy images: a multicenter study. Cornea.

[35] Üçeyler N, Vollert J, Broll B, Riediger N, Langjahr M, Saffer N, Schubert AL, Siedler G, Sommer C (2018). Sensory profiles and skin innervation of patients with painful and painless neuropathies. Pain.

[36] Devigili G, Rinaldo S, Lombardi R, Cazzato D, Marchi M, Salvi E, Eleopra R, Lauria G (2019). Diagnostic criteria for small fibre neuropathy in clinical practice and research. Brain.

[37] Terkelsen AJ, Karlsson P, Lauria G, Freeman R, Finnerup NB, Jensen TS (2017). The diagnostic challenge of small fibre neuropathy: clinical presentations, evaluations, and causes. Lancet Neurol.

[38] Hoitsma E, De Vries J, Drent M (2011). The small fiber neuropathy screening list: construction and cross-validation in sarcoidosis. Respir Med.

[39] Hobson-Webb LD, Proia AD, Thurberg BL, Banugaria S, Prater SN, Kishnani PS (2012). Autopsy findings in late-onset Pompe disease: a case report and systematic review of the literature. Mol Genet Metab.

[40] Jiang MS, Yuan Y, Gu ZX, Zhuang SL: Corneal confocal microscopy for assessment of diabetic peripheral neuropathy: a meta-analysis. Br J Ophthalmol. 2016;100 10.1136/bjophthalmol-2014-306038 25677672

[41] Bucher F, Schneider C, Blau T, Cursiefen C, Fink GR, Lehmann HC, Heindl LM (2015). Small-fiber neuropathy is associated with corneal nerve and dendritic cell alterations: an in vivo confocal microscopy study. Cornea.

[42] Ferdousi M, Azmi S, Petropoulos IN, Fadavi H, Ponirakis G, Marshall A, Tavakoli M, Malik I, Mansoor W, Malik RA (2015). Corneal confocal microscopy detects small fibre neuropathy in patients with upper gastrointestinal cancer and nerve regeneration in chemotherapy induced peripheral neuropathy. PLoS ONE.

[43] Kemp HI, Petropoulos IN, Rice ASC, Vollert J, Maier C, Strum D, Schargus M, Peto T, Hau S, Chopra R (2017). Use of corneal confocal microscopy to evaluate small nerve fibers in patients with human immunodeficiency virus. JAMA Ophthalmol.

[44] Tavakoli M, Marshall A, Thompson L, Kenny M, Waldek S, Efron N, Malik RA (2009). Corneal confocal microscopy: a novel noninvasive means to diagnose neuropathy in patients with Fabry disease. Muscle Nerve.

[45] Erkan Turan K, Kocabeyoglu S, Bekircan-Kurt CE, Bezci F, Erdem-Ozdamar S, Irkec M. Ocular surface alterations and in vivo confocal microscopic characteristics of corneas in patients with myasthenia gravis. Eur J Ophthalmol. 2018;541–546. 10.1177/1120672117753688 29554816

[46] Pena LD, Proia AD, Kishnani PS (2015). Postmortem findings and clinical correlates in individuals with infantile-onset pompe disease. JIMD reports.

[47] Winkel LP, Kamphoven JH, van den Hout HJ, Severijnen LA, van Doorn PA, Reuser AJ, van der Ploeg AT (2003). Morphological changes in muscle tissue of patients with infantile Pompe's disease receiving enzyme replacement therapy. Muscle Nerve.

[48] Nolano M, Provitera V, Manganelli F, Iodice R, Caporaso G, Stancanelli A, Marinou K, Lanzillo B, Santoro L, Mora G (2017). Non-motor involvement in amyotrophic lateral sclerosis: new insight from nerve and vessel analysis in skin biopsy. Neuropathol Appl Neurobiol.

[49] Katona I, Weis J, Hanisch F (2014). Glycogenosome accumulation in the arrector pili muscle in Pompe disease. Orphanet J Rare Dis.

[50] Falk DJ, Todd AG, Lee S, Soustek MS, ElMallah MK, Fuller DD, Notterpek L, Byrne BJ (2015). Peripheral nerve and neuromuscular junction pathology in Pompe disease. Hum Mol Genet.

[51] Sidman RL, Taksir T, Fidler J, Zhao M, Dodge JC, Passini MA, Raben N, Thurberg BL, Cheng SH, Shihabuddin LS (2008). Temporal neuropathologic and behavioral phenotype of 6neo/6neo Pompe disease mice. J Neuropathol Exp Neurol.

[52] Finnerup NB, Haroutounian S, Kamerman P, Baron R, Bennett DLH, Bouhassira D, Cruccu G, Freeman R, Hansson P, Nurmikko T (2016). Neuropathic pain: an updated grading system for research and clinical practice. Pain.

[53] Devigili G, Cazzato D, Lauria G (2020). Clinical diagnosis and management of small fiber neuropathy: an update on best practice. Expert Rev Neurother.

[54] Egenolf N, Altenschildesche CMz, Kreß L, Eggermann K, Namer B, Gross F, Klitsch A, Malzacher T, Kampik D, Malik RA (2021). Diagnosing small fiber neuropathy in clinical practice: a deep phenotyping study. Ther Adv Neurol Disord.

