# Supplementary material for: Small fiber involvement is independent from clinical pain in late-onset Pompe disease
Source: Orphanet J Rare Dis. 2022 Apr 27;17:177. doi: 10.1186/s13023-022-02327-4 (PMC9044713; doi:10.1186/s13023-022-02327-4)
Supplement: Supplementary file 1 — Additional file 1: Table S1: Clinical and demographic findings in 35 patients with LOPD [file 13023_2022_2327_MOESM1_ESM.docx]

Supplemental Table 1: Clinical findings of patients with LOPD

| **P** | **Genetic** | **GAA residual enzyme activity** | **Tissue GAA residual activity-reduction%** | **Age at skin biopsy (y)** | **Symptoms onset (y)** | **Symptoms**  **duration (symptoms onset /age at biopsy)(y)** | **Disease**  **diagnosis (y)** | **Symptoms/diagnosis (y)** | **ERT onset (y)** | **ERT duration at age at biopsy (y)** | **ERT**  **duration from symptoms onset (y)** | **Risk factor for SFN/PNP** |
| --- | --- | --- | --- | --- | --- | --- | --- | --- | --- | --- | --- | --- |
| 1 | c.719C>T; c.877G>A | - | - | 44 | 27 | 17 | 33 | 6 | 34 | 10 | 7 |  |
| 2 | c.-32-13T>G; c.1942G>A | 0.100nmol/min/mg protein | 92  (muscle) | 70 | 45 | 25 | 53 | 8 | 61 | 9 | 8 |  |
| 3 | c.-45T>G;  c.-45T>G | 0,1 U/g NCP | 96 | 46 | 31 | 15 | 40 | 9 | 40 | 6 | 9 |  |
| 4 | c.-32-13T>G; c.1716C>G | 1,5µmol//l/h | 55 | 30 | 27 | 3 | 27 | 0 | 27 | 3 | 0 |  |
| 5 | - | - | - | 54 | 45 | 9 | 45 | 0 | 54 | 0 | 9 |  |
| 6 | c.-32-13T>G; c.2608C>T | 0.25 µmol/L/h  ratio 0.08 µmol/L/h | 95 | 54 | 42 | 12 | 53 | 11 | 53 | 1 | 1 |  |
| 7 | c.-45T>G; c.877G>A | - | - | 36 | 24 | 12 | 27 | 3 | 28 | 6 | 8 | frequent alcohol intake |
| 8 | c.-32-13T>G; c.258dupC | 0.52 µmol/L/h ratio: 0.30 | 89 | 62 | 39 | 23 | 50 | 11 | 50 | 12 | 11 |  |
| 9 | c-.32-13T>G; c.1548G>A | 0.56 µmol/L/h ratio: 0.21 | 88 | 49 | 35 | 14 | 38 | 3 | 38 | 11 | 3 |  |
| 10 | c.-32-13T>G; c.1548G>A | 1.08 µmol/L/h  ratio 0.41 µmol/L/h | 78 | 48 | 41 | 7 | 41 | 0 | 41 | 7 | 0 |  |
| 11 | c.-32-13T>G; c.1710C>G,c.1923G>A | 1.30 µmol/L/h ratio: 0.29 | 73 | 72 | 28 | 44 | 61 | 33 | 61 | 11 | 33 | DMtype2 |
| 12 | c.-32-13T>G; c.525delT | <0.10 µmol/L/h  ratio <0.10 | 98 | 40 | 27 | 13 | 32 | 5 | 32 | 8 | 5 |  |
| 13 | c.-32-13T>G; c.482_483delC | < 0.01 µmol/L/h ratio < 0.01 µmol/L/h | 99 | 44 | 17 | 27 | 35 | 18 | 35 | 9 | 18 |  |
| 14 | c.-32-13T>G  p. C103G | 0,01nmol/min  (DBS) | 89 | 60 | 45 | 15 | 47 | 2 | 48 | 12 | 3 | DMtype2 |
| 15 | c.-32-13T>G; c.258dupC | <0.10 µmol/L/h ratio <0.10 µmol/L/h | 98 | 63 | 51 | 12 | 53 | 2 | 55 | 8 | 4 |  |
| 16 | c.-32-13T>G; del exon 18 | 0.29 µmol/L/h ratio: 0.10 | 94 | 62 | 44 | 18 | 52 | 8 | 52 | 10 | 8 |  |
| 17 | c.-32-13T>G; c.186dup11 | - | - | 62 | 25 | 37 | 54 | 29 | 54 | 7 | 29 |  |
| 18 | c-.32-13T>G; 525delT | 0,3µmol/l/h | 91 | 53 | 25 | 28 | 52 | 23 | 52 | 1 | 29 |  |
| 19 | c.-32-13T>G; 877G>A | 0.3µmol//l/h | 91 | 42 | 27 | 15 | 41 | 14 | 41 | 1 | 14 |  |
| 20 | c.-32-13T>G,  c. 1655T>C (p.Leu552Pro) | 0.3µmol//l/h | 91 | 49 | 46 | 3 | 48 | 2 | 49 | 0 | 3 |  |
| 21 | c.-32-13T>G; c.242delA | 0.3µmol//l/h | 91 | 74 | 72 | 2 | 74 | 2 | 74 | 0 | 2 |  |
| 22 | c. 45 T > G; c. 1051delG | - | - | 73 | 65 | 8 | 65 | 0 | 64 | 9 | 1 | Cobalamin deficiency |
| 23 | c.-32-13T>G; | - | - | 29 | 4 | 25 | 4 | 0 | 16 | 13 | 12 |  |
| 24 | c.-32-13T>G; c.1465G>A (pAsp489Asn) | 0.5µmol//l/h | 85 | 69 | 65 | 4 | 66 | 1 | 66 | 3 | 1 |  |
| 25 | c.-32-13T>G; c.2501_2502del | - | - | 34 | 3 | 31 | 24 | 13 | 24 | 10 | 11 |  |
| 26 | c-32-13T>G; c.525delT | - | - | 70 | 55 | 15 | 66 | 11 | 68 | 2 | 53 |  |
| 27 | c.32-13T>G; c.1655T>C (p.Leu552Pro) | 0.49nmol/spot/21h | 92 | 53 | 35 | 18 | 51 | 16 | 51 | 2 | 16 |  |
| 28 | c.32-13T>G; c.525delT | - | - | 39 | 30 | 9 | 37 | 7 | 37 | 2 | 7 |  |
| 29 | c.-32-13T>G;  del Exon 18 | - | - | 51 | 36 | 15 | 31 | 5 | 38 | 13 | 2 | Ferritin deficiency |
| 30 | c.32-13T>G;  c.307T>G | - | - | 45 | 36 | 9 | 42 | 6 | 42 | 3 | 6 | DMtype2 |
| 31 | c.-32-13T>G; | 6.64 nmol /min/gww | 96  (muscle) | 53 | 30 | 23 | 35 | 5 | 40 | 13 | 10 |  |
| 32 | c.32-13T>G;  2261dupC | 0.5µmol//l/h | 85 | 48 | 46 | 2 | 47 | 1 | 47 | 1 | 1 |  |
| 33 | c.32-13T>G;  c.525delT | - | - | 30 | 29 | 1 | 30 | 1 | 30 | 0 | 1 |  |
| 34 | c-32-13 T>G; c. 2237 G>A | - | - | 36 | 20 | 9 | 27 | 7 | 27 | 9 | 0 | Cobalamin and ferritin deficiency |
| 35 | c.-32-13T>G; c.1564 G>A | 0,016 mU/mg | 98 | 18 | 4 | 14 | 5 | 1 | 4 | 14 | 0 | alcohol |

GAA= acid alpha glucosidase; DM= diabetes mellitus; y= years; m=male; f=female; PNP= Polyneuropathy
