# Supplementary material for: Small fiber involvement is independent from clinical pain in late-onset Pompe disease
Source: Orphanet J Rare Dis. 2022 Apr 27;17:177. doi: 10.1186/s13023-022-02327-4 (PMC9044713; doi:10.1186/s13023-022-02327-4)
Supplement: Supplementary file 2 — Additional file 2: Table S2: Self-reported data on pain, anxiety and depression symptoms in 35 patients with LOPD [file 13023_2022_2327_MOESM2_ESM.docx]

Supplemental Table 2: Characteristics of pain of patients with LOPD

| **P** | **Gender** | **Age**  **(years)** | **Current pain intensity (PDQ1)** | **Max. pain intensity (PDQ2)** | **Mean pain intensity (PDQ3)** | **PDQ score** | **PDQ evaluation** | **HADS-A score** | **HADS-D score** |
| --- | --- | --- | --- | --- | --- | --- | --- | --- | --- |
| 1 | f | 44 | pain-free | pain-free | pain-free | pain-free | pain-free | 2 | 2 |
| 2 | m | 79 | 5 | 7 | 6 | 18 | unclear | 8 | 9 |
| 3 | f | 47 | 2 | 5 | 2 | 1 | negative | 0 | 1 |
| 4 | m | 31 | pain-free | pain-free | pain-free | pain-free | pain-free | 2 | 0 |
| 5 | m | 54 | 0 | 3 | 2 | 3 | negative | 2 | 3 |
| 6 | m | 54 | 0 | 3 | 1 | 6 | negative | 2 | 1 |
| 7* | m | 36 | 5 | 9 | 7 | 17 | unclear | 12 | 9 |
| 8 | m | 62 | pain-free | pain-free | pain-free | pain-free | pain-free | 1 | 1 |
| 9 | f | 49 | 3 | 7 | 5 | 17 | unclear | 10 | 11 |
| 10 | f | 48 | 2 | 6 | 3 | 12 | negative | 8 | 7 |
| 11* | f | 72 | 7 | 9 | 7 | 15 | unclear | 10 | 12 |
| 12 | f | 40 | pain-free | pain-free | pain-free | pain-free | pain-free | 1 | 1 |
| 13 | m | 44 | pain-free | pain-free | pain-free | pain-free | pain-free | 3 | 2 |
| 14* | m | 61 | 4 | 8 | 7 | 12 | negative | 10 | 9 |
| 15 | f | 63 | pain-free | pain-free | pain-free | pain-free | pain-free | 0 | 1 |
| 16 | f | 62 | 0 | 6 | 2 | 9 | negative | 3 | 5 |
| 17 | f | 62 | 1 | 2 | 1 | 2 | negative | 7 | 3 |
| 18 | m | 53 | pain-free | pain-free | pain-free | pain-free | pain-free | 2 | 4 |
| 19# | f | 42 | 5 | 10 | 7 | 33 | positive | 9 | 7 |
| 20 | f | 49 | 0 | 5 | 3 | 12 | negative | 3 | 1 |
| 21 | f | 74 | pain-free | pain-free | pain-free | pain-free | pain-free | 2 | 3 |
| 22* | f | 73 | 5 | 6 | 5 | 0 | negative | 13 | 17 |
| 23 | m | 29 | 0 | 2 | 1 | 1 | negative | 1 | 2 |
| 24 | f | 69 | 3 | 4 | 3 | 1 | negative | 2 | 1 |
| 25 | f | 34 | 2 | 6 | 5 | 13 | unclear | 8 | 0 |
| 26 | m | 70 | 3 | 7 | 2 | 16 | unclear | 2 | 2 |
| 27 | m | 53 | 7 | 9 | 6 | 14 | unclear | 6 | 3 |
| 28 | m | 39 | 1 | 7 | 3 | 5 | negative | 0 | 1 |
| 29* | f | 51 | 0 | 6 | 6 | 6 | negative | 3 | 2 |
| 30* | m | 45 | 0 | 7 | 5 | 3 | negative | 7 | 8 |
| 31 | f | 53 | 9 | 8 | 5 | 11 | negative | 3 | 6 |
| 32 | f | 48 | pain-free | pain-free | pain-free | pain-free | pain-free | 9 | 8 |
| 33 | f | 30 | 3 | 7 | 4 | 5 | negative | 8 | 5 |
| 34* | m | 36 | pain-free | pain-free | pain-free | pain-free | pain-free | 2 | 1 |
| 35* | m | 18 | pain-free | pain-free | pain-free | pain-free | pain-free | 0 | 0 |

M=male; f=female; PDQ= pain detection questioner; HADS = Hospitality Anxiety and Depression Scale; *=PNP risk; #=patient with neuropathic pain
