# Supplementary material for: Small fiber involvement is independent from clinical pain in late-onset Pompe disease
Source: Orphanet J Rare Dis. 2022 Apr 27;17:177. doi: 10.1186/s13023-022-02327-4 (PMC9044713; doi:10.1186/s13023-022-02327-4)
Supplement: Supplementary file 3 — Additional file 3: Table S3: Morphometric analysis of skin biopsies from 35 patients with LOPD [file 13023_2022_2327_MOESM3_ESM.docx]

Supplemental Table 3: Morphometric analyses of skin biopsies from patients with LOPD

| **Patient** | **Sex** | **Age at biopsy (years)** | **Fibers/mm** | **Cutt-off value**  **5^th^ percentile (Median) *** | **Level of reduction*** | **Z-score** |
| --- | --- | --- | --- | --- | --- | --- |
| 1 | f | 44 | 6.1 | 5.7  (11.2) | normal | -1,82 |
| 2 | m | 70 | 0.8 | 2.1  (7.7) | reduced | -2,42 |
| 3 | f | 46 | 1.6 | 5.7  (11.2) | reduced | -3,42 |
| 4 | m | 30 | 5.0 | 5.2  (10.3) | reduced | -2,04 |
| 5 | m | 54 | 6.0 | 3.5  (8.9) | normal | -1,05 |
| 6 | m | 54 | 4.5 | 3.5  (8.9) | normal | -1,60 |
| 7 | m | 36 | 6.9 | 5.2  (10.3) | normal | -1,31 |
| 8 | m | 62 | 5.1 | 2,8  (8.3) | normal | -1,14 |
| 9 | f | 49 | 6.7 | 5.7  (11.2) | normal | -1,60 |
| 10 | f | 48 | 8.4 | 5.7  (11.2) | normal | -1,00 |
| 11 | f | 72 | 4.4 | 2.2  (7.6) | normal | -1,16 |
| 12 | f | 40 | 4.5 | 5.7  (11.2) | reduced | -2,39 |
| 13 | m | 44 | 2.8 | 4.4  (9.6) | reduced | -2,56 |
| 14 | m | 60 | 1.0 | 2.8  (8.3) | reduced | -2,60 |
| 15 | f | 63 | 2.0 | 3.2  (8.7) | reduced | -2,39 |
| 16 | f | 62 | 5.3 | 3.2  (8.7) | normal | -1,21 |
| 17 | f | 62 | 6.4 | 3.2  (8.7) | normal | -0,82 |
| 18 | m | 53 | 2.6 | 3.5  (8.9) | reduced | -2,29 |
| 19 | f | 42 | 2.8 | 5.7  (11.2) | reduced | -2,99 |
| 20 | f | 49 | 5.6 | 5.7  (11.2) | reduced | -2,00 |
| 21 | f | 74 | 4.5 | 2.2  (7.6) | normal | -1,13 |
| 22 | f | 73 | 1.4 | 2.2  (7.6) | reduced | -2,25 |
| 23 | m | 29 | 3.8 | 6.1  (10.9) | reduced | -2,90 |
| 24 | f | 69 | 4.2 | 3.2  (8.7) | normal | -1,60 |
| 25 | f | 34 | 2.3 | 7.1  (12.4) | reduced | -3,74 |
| 26 | m | 70 | 4.9 | 2.1  (7.7) | normal | -0,98 |
| 27 | m | 53 | 0.2 | 3.5  (8.9) | reduced | -3,16 |
| 28 | m | 39 | 5.6 | 5.2  (10.3) | normal | -1,81 |
| 29 | f | 51 | 5.8 | 4.3  (9.8) | normal | -1,43 |
| 30 | m | 45 | 2.4 | 4.4  (9.6) | reduced | -2,71 |
| 31 | f | 53 | 2.5 | 4.3  (9.8) | reduced | -2,60 |
| 32 | f | 48 | 3.9 | 5.7  (11.2) | reduced | -2,60 |
| 33 | f | 30 | 2.3 | 7.1  (12.4) | reduced | -3,74 |
| 34 | m | 36 | 3.4 | 5.2  (10.3) | reduced | -2,65 |
| 35 | m | 18 | 3.6 | 6.1  (10.9) | reduced | -2,98 |

*Compared to reference values: reduced below 0.05 quantile value respectively

zscore < -1.96 (Lauria et.al. 2010. )
