# Supplementary material for: Small fiber involvement is independent from clinical pain in late-onset Pompe disease
Source: Orphanet J Rare Dis. 2022 Apr 27;17:177. doi: 10.1186/s13023-022-02327-4 (PMC9044713; doi:10.1186/s13023-022-02327-4)
Supplement: Supplementary file 4 — Additional file 4: Table S4: Morphometric analysis of skin biopsies from twenty healthy controls [file 13023_2022_2327_MOESM4_ESM.docx]

Suppl Table 4: Morphometric analyses of skin biopsies from healthy controls

| **Patient** | **Sex** | **Age at biopsy (years)** | **Fibers/mm** | **Cut-off value**  **5^th^ percentile (Median) *** | **Level of reduction*** | **Z-score** |
| --- | --- | --- | --- | --- | --- | --- |
| C1 | w | 24 | 10.16 | 8.4  (13.5) | normal | -1,28 |
| C2 | m | 38 | 3.4 | 5.2  (10.8) | reduced | -2,59 |
| C3 | m | 24 | 6.3 | 6.1  (10.9) | normal | -1,88 |
| C4 | m | 23 | 8.26 | 6.1  (10.9) | normal | -1,08 |
| C5 | w | 26 | 9.22 | 8.4  (13.5) | normal | -1,64 |
| C6 | m | 23 | 7.4 | 6.1  (10.9) | normal | -1,43 |
| C7 | m | 24 | 11.0 | 6.1  (10.9) | normal | 0,04 |
| C8 | m | 30 | 13.9 | 5.2  (10.3) | normal | 1,38 |
| C9 | w | 57 | 4.10 | 4.3  (9.8) | reduced | -2,03 |
| C10 | w | 53 | 7.9 | 4.3  (9.8) | normal | -0,68 |
| C11 | w | 63 | 4.2 | 3.2  (8.7) | normal | -1,60 |
| C12 | m | 53 | 4.8 | 3.5  (8.9) | normal | -1,49 |
| C13 | w | 52 | 7.56 | 4.3  (9.8) | normal | -0,80 |
| C14 | w | 49 | 5.7 | 5.7  (11.2) | normal | -1,96 |
| C15 | w | 45 | 5.8 | 5.7  (11.2) | normal | -1,92 |
| C16 | w | 62 | 12.09 | 3.2  (8.7) | normal | 1,21 |
| C17 | m | 45 | 5.9 | 4.4  (9.6) | normal | -1,39 |
| C18 | w | 45 | 7.38 | 5.7  (11.2) | normal | -1,36 |
| C19 | w | 56 | 8.6 | 4.3  (9.8) | normal | -0,43 |
| C20 | w | 63 | 6.4 | 3.2  (8.7) | normal | -0,82 |

*Compared to reference values: reduced below 0.05 quantile value respectively zscore < -1.96 (Lauria et.al. 2010)
