# Supplementary figures and images for: Small fiber involvement is independent from clinical pain in late-onset Pompe disease
Source: Orphanet J Rare Dis. 2022 Apr 27;17:177. doi: 10.1186/s13023-022-02327-4 (PMC9044713; doi:10.1186/s13023-022-02327-4)

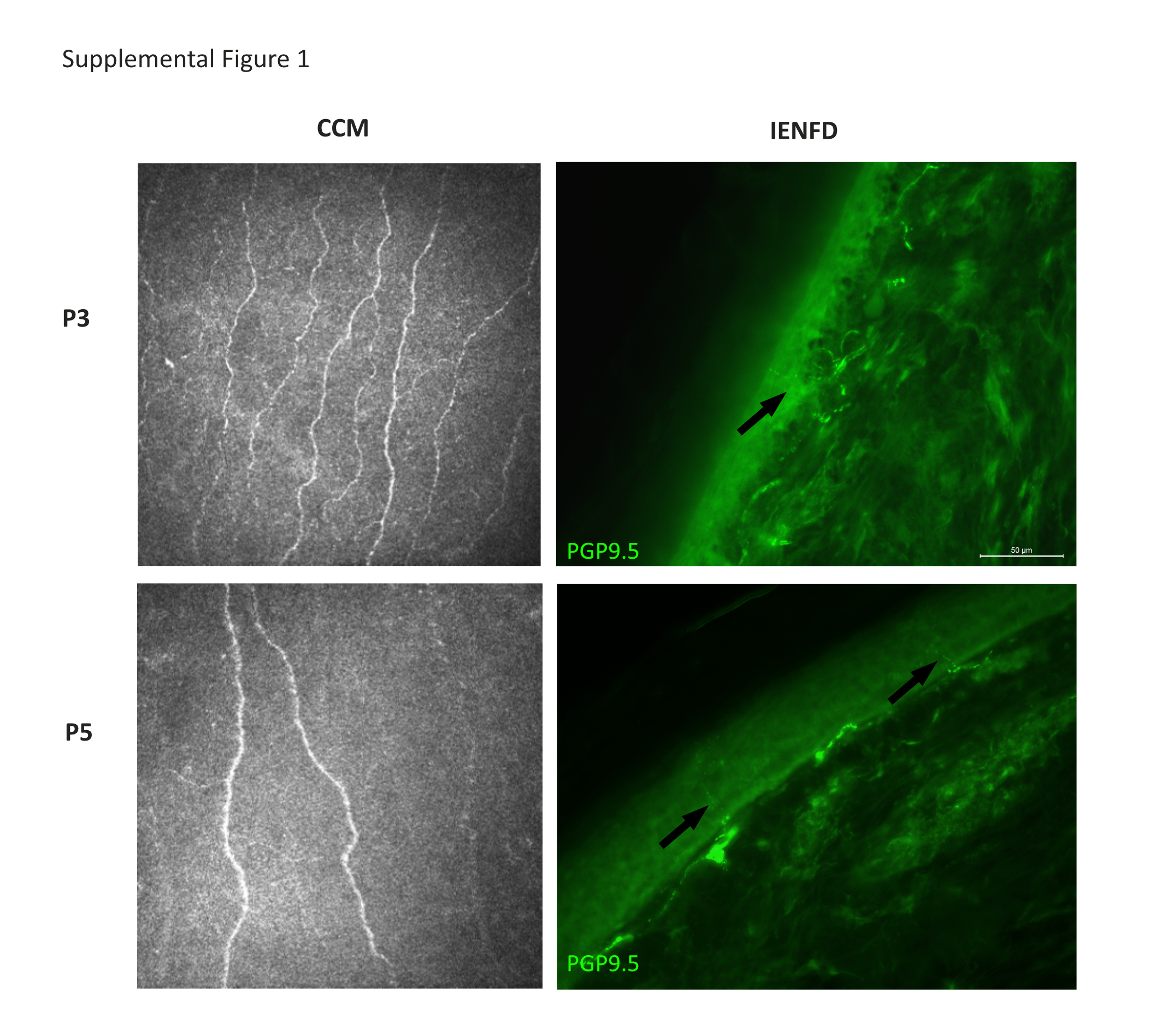

Supplement: Supplementary file 5 — Additional file 5: Fig. S1: Four patients with LOPD underwent detailed assessment of the corneal innervation by corneal confocal microscopy (CCM): While P3 showed a reduction of small nerve fibers only in the distal skin biopsy, P5 presented with abnormalities only in the corneal innervation. [file 13023_2022_2327_MOESM5_ESM.jpg]
